# Livestock-associated methicillin-resistant *Staphylococcus aureus* (LA-MRSA) prevalence in humans in close contact with animals and measures to reduce on-farm colonisation

**DOI:** 10.1186/s13620-021-00200-7

**Published:** 2021-08-06

**Authors:** Daniel Crespo-Piazuelo, Peadar G. Lawlor

**Affiliations:** Teagasc, Pig Development Department, Animal & Grassland Research & Innovation Centre, Moorepark, Fermoy, Co. Cork P61 C996 Ireland

**Keywords:** LA-MRSA, Animal production, Prevention, Antimicrobial resistance, Animal contact

## Abstract

Since the 1940s, *Staphylococcus aureus* has adapted to the use of different antimicrobials to treat infections. Although *S. aureus* can act as a commensal bacterium, some strains are facultative pathogens and acquiring them can be fatal. In particular, treating infections caused by *S. aureus* with acquired antimicrobial resistance is problematic, as their treatment is more difficult. Some of these *S. aureus* variants are methicillin-resistant *S. aureus* (MRSA) with prevalence across the globe in health-care facilities, community settings and on livestock farms. Apart from humans, MRSA can colonise other animal species, and because of this, resistance to new antimicrobials can appear and jump between species. Livestock and companion animals are particularly important in this regard considering the relatively high usage of antimicrobials in these species. There is a risk to humans who come into direct contact with animals acquiring MRSA but there is also the risk of animals acquiring MRSA from colonised humans. In this review, we summarise studies conducted worldwide to characterise the prevalence of MRSA in veterinarians, farmers and other personnel who come into close contact with animals. Finally, alternative treatment, preventive measures and on-farm strategies to reduce MRSA introduction to a farm and carriage within a herd are discussed.

## Introduction

*Staphylococcus aureus* is a Gram-positive catalase-positive bacterium which is commonly found on the skin and mucosa of humans and animals [1–3]. The anterior nares of humans are one of the most frequently colonised sites and about 30% of the human population is colonised with *S. aureus* [3, 4]. Although *S. aureus* is usually classified as a commensal bacterium, it is a facultative pathogen, which can potentially cause several diseases from mild skin lesions to severe and potentially fatal infections [5]. As a commensal bacterium, *S. aureus* colonises its host without impacting the health of the host. Depending on the persistence of carriage, hosts can be persistent carriers, intermittent carriers, or non-carriers at all, if the bacterium rarely colonises the host, and when it does, the colonisation is transient and does no last longer than about two weeks [6]. However, nasal swabs from persistent carriers usually yield the same MRSA strains over time, and because of the facultative pathogenicity of *S. aureus*, persistent carriers are at a higher risk of developing an infection [6].

Within a few years of the introduction of penicillin in the 1940s, the first cases of penicillin-resistant *S. aureus* were reported [7]. Resistance was obtained through the acquisition of plasmids that contained the ß-lactamase gene (*blaZ*), which produce an enzyme capable of breaking down the β-lactam ring of penicillin and other antibiotics [8–10]. The β-lactam ring of penicillin causes bacterial lysis by binding to penicillin-binding proteins (PBP), which are needed for cross-linking the peptidoglycan chains of the cell wall [11].

Methicillin, a new antibiotic with resistance to β-lactamase was developed two decades later, in 1960, and in less than two years, the first methicillin-resistant *S. aureus* (MRSA) appeared [12, 13]. This new resistance was driven by the acquisition of the *mecA* gene, which encodes the penicillin-binding protein 2a (PBP2a), a slightly different PBP that possesses low affinity for β-lactam antibiotics [14].

Later on, in the 1980s and 1990s, MRSA strains spread across the world carrying multidrug resistant traits and being one of the most important agents of nosocomial infection [5]. These MRSA strains were called hospital-acquired MRSA (HA-MRSA), as they were commonly found in hospitals and health care facilities.

In parallel with the spread of HA-MRSA, control measures were applied in hospital settings to prevent nosocomial transmission of MRSA [15]. These preventive measures reduced HA-MRSA prevalence in several countries. However, infections caused by new MRSA strains started to increase in communities outside the hospital setting in the 1990s [16, 17]. These strains which could spread rapidly among groups of healthy individuals were called community-acquired MRSA (CA-MRSA) [18].

In 2004, a new MRSA strain was found colonising the daughter of a pig farmer in the Netherlands [19]. Both parents and a pig on the family farm carried the same strain, which was characterized by the presence of the *mecA* gene. This strain was different from those usually found in HA-MRSA and CA-MRSA, as it was impossible to classify it using the standard method of pulsed-field gel electrophoresis (PFGE) with restriction endonuclease *SmaI*. Although other studies previously reported a linkage between animal and human MRSA colonisation, this was the first study to demonstrate the transmission of MRSA between animals and humans. Later on, several other studies reported this in other countries. The term livestock-associated MRSA (LA-MRSA) was used to refer to this third group of MRSA strains which were considered a reservoir in livestock animals [20].

There are several methods for screening and typing MRSA strains: *spa* sequence typing; multilocus sequence typing (MLST); staphylococcal cassette chromosome *mec* (*SCCmec*) typing; PFGE; and multilocus variable-number tandem repeat (VNTR) analysis (MLVA) (reviewed in [21, 22]). Based on MLST, strains are assigned to a sequence type (ST) after sequencing seven endogenous genes. Identical strains by MLST are assigned to the same ST, while strains with closely related STs may belong to the same clonal complex (CC) lineage (e.g., CC398). Another most commonly used method is *spa* typing, based on sequencing the variable X region of the *S. aureus* surface protein A (*spa*) gene and assigning the strains to a spa type based on the different polymorphisms found (e.g., t011). The last method that has received most attention is *SCCmec* typing, which sequences and classifies the strains based on the differences in mobile genetic elements (MGE) including the aforementioned *mecA* gene (e.g., *SCCmec* IV). Despite the efforts in classifying the different MRSA strains found, the epidemiology of MRSA colonisation is changing. Strains of CA-MRSA can share genes between both LA- and HA- MRSA [23], and some clones are present in more than one classification group, blurring the distinction between strains [24–26].

LA-MRSA strains can be transmitted between different animal species and to humans who come in close contact with colonised animals, such as veterinarians and farm workers. However, colonised humans can also transmit LA-MRSA to other humans and between animal settings. In this review, we have summarised studies conducted worldwide to assess the prevalence of MRSA in personnel who come into close contact with animals. We also discuss preventive measures to avoid MRSA introduction onto a farm, alternative treatments and on-farm strategies to reduce or eradicate MRSA carriage within a herd.

### MRSA transmission between humans, animal production and companion animals

MRSA can be transmitted from vertebrate animals to humans. Likewise, humans also act as a reservoir for the transmission of *S. aureus* to vertebrate animals. Infections that can be present in both humans and animals and transmitted in both directions, such as *S. aureus* infections, are defined as “amphixenoses” [27]. The first reported incident of MRSA colonisation in livestock happened in Belgium in the early 1970s, affecting the milk of cows with bovine mastitis [28]. Despite most of the isolates having been similar to common bovine strains, a human-to-animal transmission of a new MRSA strain acquired by the farmer seems more likely due to the observed antimicrobial characteristics. Since then, MRSA colonisation has been reported in dogs, cats, horses, cattle, pigs, rabbits and poultry [29–32]. It has even been described in wild birds such as magpies and vultures [33]. The first animal-to-human transmission of *S. aureus* was reported in dairy sheep [34]. Shortly after, the first case of MRSA transmission from animal to human, including human-to-human transmission of the same strain was reported [19]. Other interspecies transmission have been described in household pets, which can be colonised with the same strains as their owners [35]. Nonetheless, despite the potential of *S. aureus* to colonise domestic and livestock animals, the primary reservoirs of LA-MRSA in affected countries are those animals in intensive systems (i.e., pigs, veal calves, and broilers) [36].

*S. aureus* is usually transmitted by direct contact with colonised skin or with a mechanical vector [37]. In livestock systems, the most important ways of introducing MRSA onto a farm are the movement of MRSA colonised animals from one farm to another, direct contact with colonised humans and through animal contact with contaminated transport vehicles [38–40]. Another indirect way of transmitting MRSA between species is through the environment. *S. aureu*s has a half-life of 5 days in dust, but it can survive in it for weeks [41]. These small particles can be transported to other farms on vehicles, and farm-workers can expose their families to the bacterium by bringing home dust covered clothes. Dust can also be transported by the wind along with other substances; airborne staphylococci has been detected at 477 m from a commercial broiler barn, and up to 530 m in theory [42]. Indeed, airborne-transmission of MRSA in hospitals [43], and to personnel that work more than 20 h per week in farm barns [44] is also possible. Apart from direct contact with animals or humans in contact with them, MRSA can also be transmitted through food production systems. Animals can act as a reservoir for antimicrobial-resistant bacteria and their products, such as meat, milk and eggs, can be a means of transmission of these bacteria to the consumer [45, 46]. However, the risk of acquiring MRSA through food appears to be low [36].

Despite certain *S. aureus* strains being closely adapted to a specific host, they can be transmitted to other species [47–49]. These colonisations outside their preferred hosts are usually transient and generally do not last long [50], although some can be transmitted and sustained among the new host species [47, 49]. Moreover, MRSA strains continuously evolve in the host, and can even be replaced by other strains [51]. There are several factors that can influence MRSA transmission between animals and humans. The intensity/duration of animal contact is one such; farmers working more hours in a calf stable were more often carriers than farmers working fewer hours [50, 52]. In the absence of animal contact, LA-MRSA carriage was often reduced or lost, although some persistent carriers were reported [50]. Furthermore, the number of MRSA-positive animals within the farm also increases the probability of MRSA being transmitted to the farmer [52]. Following this trend, people living in areas with a higher livestock density (pigs, cattle, and veal calves) or in proximity to areas where pig manure is applied to crop fields are also at a higher risk of being colonised by LA-MRSA [53, 54].

### LA-MRSA colonisation in humans in close contact with animals

Most of the LA-MRSA strains that colonise humans will not necessarily cause an infection, and when they occur they are usually less severe than those caused by HA- and CA-MRSA [55]. In addition, LA-MRSA strains represent a small proportion (3.9%) of the isolated MRSA in humans in the European Union, but their proportion was higher (≥ 10%) in five countries (Belgium, Denmark, the Netherlands, Slovenia and Spain) [56]. However, every *S. aureus* strain has the potential to evolve into a life-threatening pathogen [57]. For instance, in 2014, four patients died from LA-MRSA bacteraemia in Denmark, raising the concern regarding LA-MRSA in the European Union [56]. Today, hospital anamnesis usually includes questions to target farm workers or personnel in close contact with animal production settings due to their increased risk of being colonised with MRSA.

**Fig. 1:**
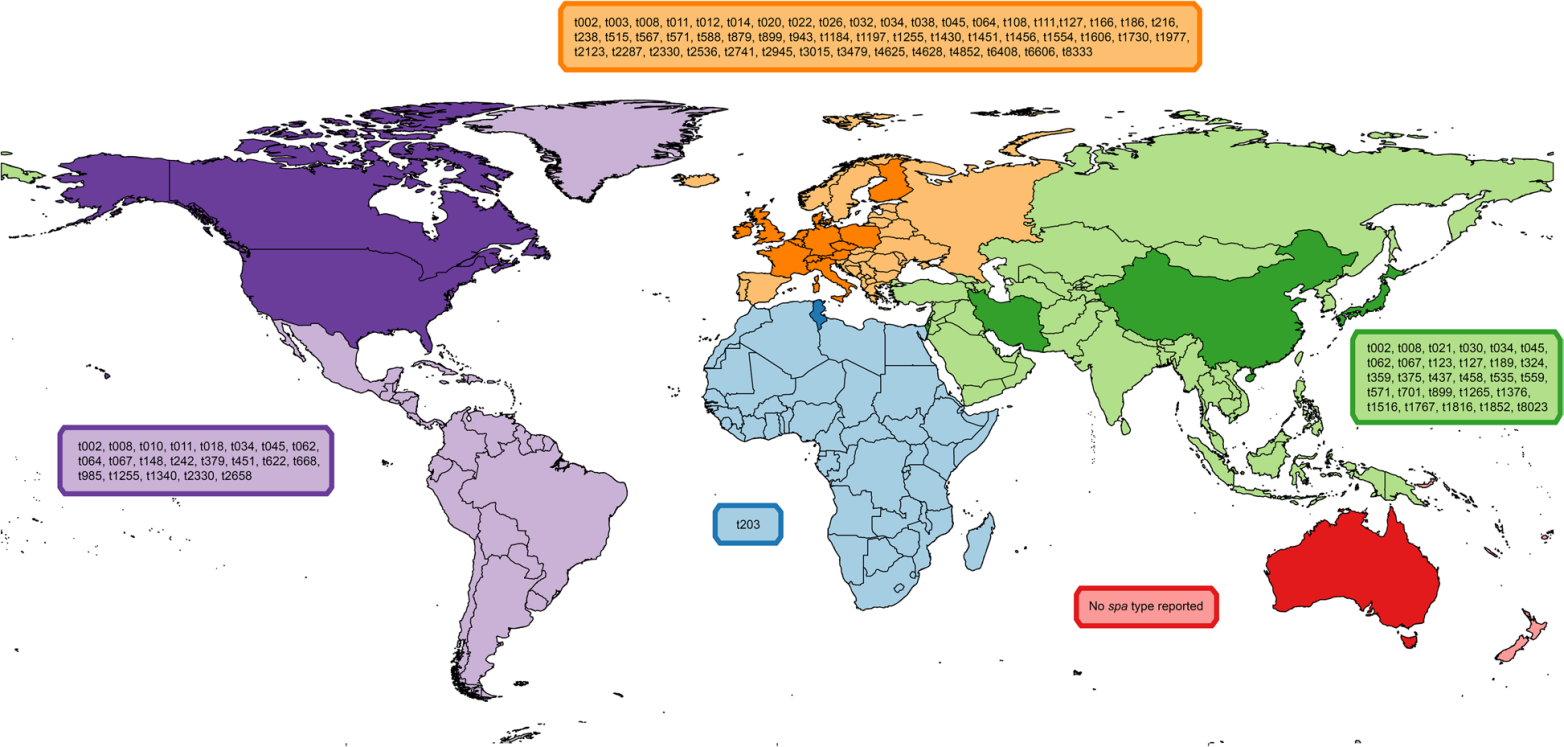
MRSA *spa* types carried by humans in close contact with animals grouped by continent. Coloured boxes enclose the *spa* types reported in the studies summarised in Table 1. Countries whose prevalence was reported in Table 1 are highlighted in a darker colour

Several studies have concluded that humans in close contact with animals have greater risk of being colonised by LA-MRSA than the rest of the population [58–62]. Although some studies have reported that veterinarians are more likely to be MRSA carriers than farmers [60, 62], meta-analyses of published data established that livestock workers are at a higher risk to be colonised with LA-MRSA, more so if they are pig farmers [63, 64]. However, colonisation in veterinarians is a potential thread for MRSA spread among farms. To assess the prevalence of MRSA colonisation in veterinarians, an NCBI Database search for the terms “veterinarian”, “prevalence” and “MRSA” was conducted. The resulting studies are summarized in Table 1, while the *spa* types carried by the humans analysed are reported in Fig. 1.

**Table 1:** Prevalence of MRSA in veterinarian personnel and animal workers reported in studies conducted worldwide

Study	Year conducted	Country	Prevalence	N	Population
(Chen et al., 2020) [65]	2019	China	31.71%	41	Veterinarians (veterinary hospitals)
(Schmidt et al., 2020) [66]	Aug 2018-Jan 2019	Switzerland	7.07%	99	Veterinary personnel from animal companion clinics
(Kittl et al., 2020) [67]	2017	Switzerland	5.13%	156	Farmers
(Kittl et al., 2020) [67]	2017	Switzerland	6.60%	212	Veterinarians
(Neradova et al., 2020) [68]	2017	Czech Republic	6.72%	134	Veterinary personnel
(Taus et al., 2019) [69]	2017	Austria	13.40%	261	Veterinarians
(Taus et al., 2019) [69]	2017	Austria	38.30%	47	Swine veterinarians
(Taus et al., 2019) [69]	2017	Austria	7.90%	214	Non-swine veterinarians
(Verkola et al., 2019) [70]	2016	Finland	0.30%	320	Veterinarians
(Tabatabaei et al., 2019) [71]	Nov 2012—Mar 2013	Iran	4.00%	50	Veterinary personnel
(Sun et al., 2017) [72]	2012	USA	9.50%	66	Swine veterinarians
(Wang et al., 2017) [61]	Nov 2013-Nov 2014	China	5.10%	335	Farmers and veterinarians (pig-related workers)
(Mroczkowska et al., 2017) [60]	Aug 2010-Nov 2012	Poland	3.20%	283	Pig farmers
(Mroczkowska et al., 2017) [60]	Aug 2010-Nov 2012	Poland	10.50%	38	Veterinarians
(Post et al., 2017) [73]	2013	Worldwide	5.00%	60	Veterinary surgeons
(Walter et al., 2016) [74]	2008–2009	Germany	9.00%	695	Veterinarian attending to different species
(Walter et al., 2016) [74]	2008–2009	Germany	8.53%	516	Veterinarian in contact with cattle
(Walter et al., 2016) [74]	2008–2009	Germany	14.52%	365	Veterinarian in contact with pigs
(Steinman et al., 2015) [75]	2012	Israel	16.90%	59	Staff members of a veterinary hospital
(Verkade et al., 2014) [76]	Jul 2008-Dec 2009	The Netherlands	44.00%	137	Veterinarians
(Wettstein Rosenkranz et al., 2014) [77]	2012	Switzerland	2.73%	146	Veterinarians of small animals
(Wettstein Rosenkranz et al., 2014) [77]	2012	Switzerland	6.45%	31	Veterinarians of large animals
(Wettstein Rosenkranz et al., 2014) [77]	2012	Switzerland	4.50%	111	Veterinarians of general practice
(Ishihara et al., 2014) [78]	2008	Japan	22.90%	96	Veterinarian for dog and cats
(Ishihara et al., 2014) [78]	2008	Japan	10.00%	70	Veterinarian technician for dog and cats
(Paterson et al., 2013) [79]	2011	UK	2.60%	307	Cattle Veterinarians (Mostly)
(Boost et al., 2013) [80]	Not specified	Hong Kong, China	5.60%	300	Pork butchers
(Schwaber et al., 2013) [81]	2010	Israel	50.00%	20	Full-time equine staff
(Schwaber et al., 2013) [81]	2010	Israel	4.55%	22	Community equine veterinarians
(Garcia-Graells et al., 2012) [82]	2010	Belgium	8.90%	105	Livestock veterinarians
(Garcia-Graells et al., 2012) [82]	2010	Denmark	2.10%	97	Livestock veterinarians
(Paul et al., 2011) [83]	2010	Italy	1.60%	128	Small animal dermatologists
(Jordan et al., 2011) [84]	2009	Australia	4.80%	250	Dog and cat veterinarians
(Jordan et al., 2011) [84]	2009	Australia	21.35%	89	Horse veterinarians
(Jordan et al., 2011) [84]	2009	Australia	8.33%	12	Pig veterinarians
(Zhang et al., 2011) [85]	2008–2009	China	1.96%	51	Small animal veterinary staff
(Horgan et al., 2011) [86]	2008	Ireland	2.00%	100	Pig Health Society Symposium attendees
(Ben Slama et al., 2011) [87]	2008–2009	Tunisia	1.20%	83	Veterinarian students or staff, farmers and abattoir workers
(Ishihara et al., 2010) [88]	2007	Japan	25.00%	20	Veterinarians
(Ishihara et al., 2010) [88]	2008	Japan	23.50%	34	Veterinarians
(Ishihara et al., 2010) [88]	2007	Japan	11.96%	92	Veterinary personnel
(Ishihara et al., 2010) [88]	2008	Japan	7.87%	127	Veterinary personnel
(Huber et al., 2010) [62]	2009	Switzerland	0.00%	148	Pig farmers
(Huber et al., 2010) [62]	2009	Switzerland	3.01%	133	Veterinarians
(Huber et al., 2010) [62]	2009	Switzerland	0.00%	179	Slaughterhouse employees
(Burstiner et al., 2010) [89]	2008	USA	17.30%	341	Veterinary surgeons and technicians
(Boost et al., 2011) [90]	Not specified	Hong Kong, China	0.67%	150	Veterinary personnel
(Heller et al., 2009) [91]	Not specified	Scotland, UK	3.13%	64	Small animal hospital staff members
(Zemlicková et al., 2009) [92]	2008	Czech Republic	0.70%	280	261 Veterinary professionals (veterinarian and technician); 19 Pharmacist
(Meemken et al., 2008) [93]	2007	Germany	23.26%	86	Veterinarians, laboratory personnel and meat inspection personnel
(Moodley et al., 2008) [94]	Aug 2006-Feb 2007	Denmark	3.90%	231	Veterinarians (small and large animals)
(Moodley et al., 2008) [94]	Aug 2006-Feb 2008	Denmark	0.00%	98	Farmers

Prevalence of MRSA carriage in the general human population has been reported to be in the range of 0.8–1.3% [79]. When the data presented in Table 1 relating to veterinary personnel is considered, the prevalence reported ranged between 0 and 50% with an overall mean prevalence of 8% across studies, as most studies described a prevalence less than 10%. Most of the studies considered in this review were conducted in Europe and the most commonly reported strain was CC398 (usually *spa* type t011), considered the dominant LA-MRSA strain in Europe. Some other strains such as CC5 (usually *spa* type t002) were reported as more dominant in Asia. HA-MRSA and CA-MRSA strains were also reported in these studies, making it difficult to discern the host preference of MRSA strains. Despite CC398 being considered the classical LA-MRSA strain, it originated in humans as methicillin-susceptible *S. aureus* [95]. Nowadays, the transmission of CC398 from animals to humans is considered difficult, as this strain does not usually persist in humans [50]. Nonetheless, this paradigm is changing and fatal cases of infection in humans with CC398 have been reported [56]. Other strains of MRSA, such as CC97 which is transmitted between pigs and cattle in Italy, affect more than one animal species [96]. In addition, colonisation with MRSA is dependent on the duration of animal contact and the strain involved [50]. In this sense, farm workers who are frequently in close contact with animals are at a higher risk of acquiring MRSA, even more so than veterinarians whose duties include drug administration and necropsies. The age of the animals is also considered a risk factor. Younger pigs are more likely to be colonized with MRSA [64] and it is interesting that these younger pigs generally require the most intensive handling and care.

The higher prevalence of MRSA colonisation in veterinarians from countries such as the Netherlands, Germany and USA can be explained by their having large pig herd sizes and intensive production systems. Although having smaller herd sizes, Austria also reported a higher prevalence of MRSA colonisation in swine veterinarians compared with non-swine veterinarians. However, veterinary personnel from clinics and hospitals for small animals or horses also showed a high prevalence of MRSA colonisation worldwide. A study conducted in 2008 in Ireland reported a low prevalence of MRSA colonisation in pig industry personnel, and none of the samples found were CC398 MRSA [86]. After the first reported case of CC398 MRSA (*spa* t011) in 2012, other incidents of CC398 MRSA colonisation have been reported on two Irish farms and in a veterinarian [97]. This demonstrates that natural barriers, such as Ireland’s island status, are not sufficient in preventing MRSA spread unless they go in hand with a surveillance program which controls animal importations and strict on-farm biosecurity measures for personnel visiting or returning from abroad. Switzerland also reported a low prevalence for MRSA colonisation among veterinarians, pig farmers and slaughterhouse employees in 2009. Over a decade later, the prevalence in veterinarians has increased along with the prevalence of MRSA in Swiss livestock [67], despite ongoing efforts to reduce antimicrobial usage [64]. This increase seems to be due to the spread of *spa* t034 among the swine herd there. The prevalence of MRSA in the Swiss pig population further increased with the rapid spread of *spa* t011, but this strain seems to be poorly adapted to colonising humans. Swiss cattle are also colonised by MRSA, but its increase in prevalence has been slower. As the importation of pigs into Switzerland is low and combined with the fact that many farms there have mixed livestock enterprises (pigs, cattle, poultry and/or horses) could suggest that another species other than pigs may have acted as a reservoir of MRSA there.

### Shouldn’t reduced antimicrobial usage decrease the prevalence of MRSA in farm settings?

Most European countries reduced antimicrobial consumption in the period from 2010 to 2017 [98]. However, the prevalence of MRSA in animals has not decreased in these countries, but rather, in some (Finland, Spain and Switzerland) it has increased while in others it has remained stable (Germany and Norway) [99]. A possible explanation for this could be that once MRSA becomes endemic in a setting, it is difficult for other bacteria to compete and displace resistant strains. The selective pressure of antibiotic use allows MRSA to thrive, whereas it is more difficult for the non-resistant bacteria to survive. In theory, if antibiotics are no longer used, other susceptible strains of *S. aureus* or other bacteria should out-compete MRSA due to the fitness cost of expressing the resistance genes. However, some MRSA strains possess resistance genes with low fitness-costs that allow them to compete and dominate hospital settings [100]. In a controlled environment, such as a hospital setting, reduced antimicrobial consumption in humans has proven effective in reducing MRSA presence [101], but these strategies do not necessarily decrease bacterial resistance [102]. Along with reducing antimicrobial use, what could be a more effective strategy for reducing MRSA levels in a setting is to frequently change the class of the antibiotics used [102, 103]. In this scenario, MRSA will not have a selective advantage over methicillin-susceptible strains if both are susceptible or resistant to the same prescribed antibiotic, and due to competition, susceptible strains will increase in circulation in detriment of MRSA. MRSA can also revert spontaneously to a susceptible strain if there is no selective pressure from antibiotics [104]. Nonetheless, if only the MRSA strain is resistant to the prescribed antibiotic, MRSA circulation will be enhanced. This frequent change in the class of antibiotic treatment has the potential to eliminate the bacterium, but it also has the risk of selecting for multi-drug resistance if treated strains are susceptible, as some of them may develop new resistances and survive the next change in antibiotic, accumulating resistance.

Another possible explanation as to why MRSA is increasing in prevalence on livestock farms is that humans can act as a reservoir for MRSA [105]. Research usually focuses on MRSA strains that are transmitted from animals to humans and the duration of the colonisation. However, colonised humans are an important source for introducing MRSA onto the farm setting [106]. As an example, one of the outbreaks of MRSA in Norway happened through the introduction onto farms of CC1 MRSA, described mainly as an HA-MRSA, by a farm worker and spread through animal trading between farms [25]. Although CC1 has been described mainly as an HA-MRSA, it is not uncommon to find it in animals [107]. Therefore, we consider that the study of the transmission of MRSA from humans to animals should receive more attention, as it is likely that the spread of MRSA in livestock within and across countries may be due to animal contact with infected farm workers and/or veterinarians.

### Alternative treatments for MRSA colonisation in animals

There is increasing concern worldwide regarding antimicrobial resistance (AMR) because fewer options for treating immunocompromised patients will be available if AMR is not reduced. In 2016, the United Nations (UN) General Assembly discussed strategies for fighting AMR from a joint perspective (www.un.org/pga/70/events/high-level-meeting-on-antimicrobial-resistance/). One of the greatest challenges that human health faces is the appearance of bacteria resistant to last resort antibiotics for humans and livestock species, such as vancomycin-resistant *S. aureus* [108, 109]. It is important to note that some of the strategies to avoid the appearance of these new resistances include the elimination of last resort drugs from use in animal production. This policy is being applied in China, the main consumer of antimicrobials for veterinary use, although some European countries still retain the use of these drugs in farm settings [110]. Alongside the need to reduce antibiotic usage, there has been an increase in the number of studies looking for alternative treatments to control/prevent MRSA colonisation.

Probiotics are one of the alternatives to antibiotics for the treatment and control of MRSA that have received most attention. The abundance of certain bacteria, such as lactic acid bacteria (*Lactobacillus* spp.), is negatively correlated with *S. aureus* abundance [111], which suggests that these bacteria may inhibit MRSA growth. The production of hydrogen peroxide (H_2_O_2_) by vaginal lactobacilli has been suggested as a possible bacteriostatic mechanism for reducing *S. aureus* levels [112, 113]. However, the low concentrations of O_2_ found in the vaginal environment, which is required for H_2_O_2_ production, suggests that the antimicrobial properties of *Lactobacillus* spp. may be linked to production of another substance (e.g. lactic acid) which they produce in hypoxic environments [114]. Another mechanism in which *Lactobacillus* spp. might inhibit *S. aureus* colonisation is through the secretion of a biosurfactant that impedes the adhesion of *S. aureus* to surfaces [115]. Although most of the studies concerning the use of bacteria to control *S. aureus* have been performed in vitro, some have been conducted in vivo in mice and humans. The oral administration of *Lactobacillus* spp. reduced *S. aureus* carriage in the gastrointestinal tract of humans [116]. Whether consumed orally or administered in conjunction with nasal sprays, lactobacilli have also been used with the purpose of eradicating long-term nasal carriage of MRSA in humans. The mode of action of the lactobacilli here is thought to be mediated through stimulation of the upper respiratory immune system of the host. Nonetheless, such treatments have not been completely effective [117, 118].

With the global trend towards reducing antimicrobial usage in livestock, the use of probiotics in food-producing animals seems a viable alternative. The addition of probiotics, such as *Bacillus subtilis*, to feed can improve feed efficiency in pigs whilst these probiotics also possess in vitro inhibitory activity over *S. aureus* [119]. The action of *B. subtilis* against *S. aureus* is mediated through the use of fengycins, a lipopeptide that inhibits quorum sensing in *S. aureus* colonies, impairing their ability to perceive their population density and act in response with genetic adaptations [120]. In staphylococcal infections, quorum sensing regulates, among others, toxin production, the expression of colonisation factors and biofilm formation [121]. Of note, the presence of *B. subtillis* in the human intestine was associated with the absence of *S. aureus* not only in the intestine, but also in the nasal passage, even though *B. subtilis* was seldom found colonising the airways of subjects [120].

Bacteria from various environments have been evaluated to ‘mine’ new probiotics with bacteriostatic activity against MRSA. For example, in an Irish study, *Streptomyces* sp. myrophorea recovered from soil has been reported to have activity against several pathogens, including MRSA [122]. Other approaches have studied the microbial ecology of the host using 16S rRNA gene sequencing and considered the co-abundance of the bacteria present to analyse those species positively and negatively correlated with MRSA. In one such study, *S. aureus* colonisation was seldom found in the nasal passages of pigs when other staphylococci species (*S. sciuri*, *S. cohnii*, and *S. saprophyticus*) were present [123].

Another possible alternative for reducing MRSA carriage is the use of bacteriophage therapies. The treatment of staphylococcal skin lesions was the target of one of the first bacteriophage therapies used [124]. However, phage therapies were relegated to a second place in Western medicine a few decades later because of the wide-spread use of antibiotics [125]. In recent years, phage therapy research has again resurfaced to find alternatives to antimicrobial treatments. Clinical trials in mice have shown that bacteriophage administration can protect against lethal MRSA infections and reduce *S. aureus* levels in the nasal passages [126, 127]. Although promising, clinical trials with bacteriophage therapies are still rare due to a number of challenges, including the possibility of MRSA acquiring resistance to the phage, side effects of bacterial lysis, governmental constraints and public reticence towards administration of a self-replicating agent [125, 128].

### Eradication strategies

MRSA can be eradicated from hospitals by implementing strict hygiene measures and controlling the environment. For instance, ‘search and destroy’ policies were applied in the Netherlands and Denmark reducing the spread of both HA- and CA-MRSA in health-care settings [129, 130]. In these strategies, screening for MRSA carriage is performed on patients and health-care workers. Thereafter, all the individuals positive for MRSA are isolated and treated, when possible, in order to eliminate MRSA carriage. Similar preventive measures have been applied in the U.S., such as in the REDUCE-MRSA trial, which reported a 37% reduction of MRSA carriage when universal decolonisation was practiced on all patients in the same intensive care units (ICUs) through intranasal mupirocin application and bathing with cloths impregnated in chlorhexidine [131]. Measures for the prevention and control of MRSA infections and the interruption of its transmission in U.S. hospital settings were successful in reducing the incidence of HA-MRSA bacteraemia in the last few decades [132]. However, the rate of decline in HA-MRSA infections has slowed since 2012 in the U.S., while at the same time the incidence of CA-MRSA infections has increased slightly. This suggests the need for new preventive measures [132].

In 2013, Norway commenced a national control strategy to eradicate MRSA from pig farms [40]. The strategy involves the annual screening of all pig herds but also a surveillance program of the human population, following the “One Health” approach. Information from reported human MRSA cases in hospitals allows the tracing of MRSA spread across Norway. Furthermore, introduction of MRSA into pig herds in Norway from abroad can easily be traced since Norwegian pig production is a particularly closed system, with few stock importations [40]. The Norwegian eradication campaign follows a series of steps, starting with an annual screening of the pig population. When a farm is found to be positive for MRSA, trade in live animals is restricted, and farms are depopulated followed by thorough washing and disinfection. Once confirmed free of MRSA, the farm is re-stocked with pigs from MRSA negative herds. Even then, animals from the restocked farm are screened again before reaching the slaughterhouse to assess the success of MRSA eradication [40]. As part of the programme, farm staff and their relatives are screened for MRSA carriage. There are increased restrictions on visiting Norwegian pig farms, especially if the visitor is from abroad, to help limit potential colonisation. It would seem that this strict, expensive and time-consuming eradication programme is effective as the prevalence of MRSA in Norwegian pigs has been kept low (< 0.5%) [99].

### Suggestions to prevent MRSA introduction onto farms

The introduction of MRSA onto a farm can occur via different routes. The most important of these being the movement of MRSA colonised animals from one farm to another, direct contact with colonised humans and through animal contact with contaminated transport vehicles [38–40]. For this reason, good external biosecurity protocols should be adhered to when importing live animals onto a farm. Stock to be introduced should only be purchased from MRSA-negative herds. They should be quarantined at 550 m from the rest of the herd for a minimum period of 6 weeks as it has been shown that MRSA can be airborne and spread in this manner [42, 43, 133]. Animals should be screened for MRSA during quarantine and only allowed join the main herd when shown to be MRSA-negative. Gloves should be worn when animals are handled and all humans working or visiting the farm should shower in before entering and shower out when exiting the farm [36]. The number of visitors entering the farm should be kept to a minimum, but when necessary, visitors should not have been on another farm in the previous 48 h as LA-MRSA can still be carried during that period [134]. Furthermore, farm visitors should wear masks to reduce transmission to the herd [135]. Meat products should not be permitted on the farm, as MRSA can also survive in them [45, 46].

Antimicrobial use on farms should be minimised. When MRSA first enters a farm, antimicrobial use helps to select for MRSA strains, to the detriment of the non-resistant strains [39]. In a model to assess the efficacy of interventions on the spread of MRSA on Danish pig farms, reducing antimicrobial use, restricting movement of animals and reducing human transmission were predicted to be highly effective when introduced in a low MRSA prevalence area [106]. However, these measures need to be implemented before an endemic situation occurs, otherwise much stricter control measures, like those in Norway, will be required, such as culling a percentage of MRSA positive herds.

## Conclusion

Despite reduced antimicrobial usage in European animal production in recent years, the prevalence of MRSA in farm animals has not declined. It is therefore likely that, MRSA persists in other reservoirs, such as in humans (i.e. farm workers and veterinarians). In highly colonised farms, strategies involving reducing the reliance on antibiotic usage and the frequent change of antibiotic classes used should be considered for reducing MRSA levels. Control and treatment strategies such as, strict preventive biosecurity measures, selective probiotic feeding and MRSA eradication programmes should be used to prevent MRSA entering a farm, reduce MRSA carriage and eradicate MRSA from infected farms, respectively. Doing so will help to prevent a future where antibiotics are no longer an effective treatment for MRSA infections in animals and humans.

## Data Availability

Not applicable.

## Abbreviations

AMR: Antimicrobial resistance
*blaZ*: ß-lactamase gene
CA-MRSA: Community-acquired MRSA
CC: Clonal complex
H_2_O_2_: Hydrogen peroxide
HA-MRSA: Hospital-acquired MRSA
ICUs: Intensive care units
LA-MRSA: Livestock-associated MRSA
MGE: Mobile genetic elements
MLST: Multilocus sequence typing
MLVA: Multilocus VNTR analysis
MRSA: Methicillin-resistant *S. aureus*
PBP: Penicillin-binding proteins
PBP2a: Penicillin-binding protein 2a
PFGE: Pulsed-field gel electrophoresis
*SCCmec*: Staphylococcal cassette chromosome *mec*
*spa*: *S. aureus* surface protein A
ST: Sequence type
UN: United Nations
VNTR: Variable-number tandem repeat
